# Using a participatory design to develop an implementation framework for integrating falls prevention for older people within the Chinese primary health care system

**DOI:** 10.1186/s12877-024-04754-3

**Published:** 2024-02-21

**Authors:** Pengpeng Ye, Junyi Peng, Ye Jin, Leilei Duan, Yao Yao, Rebecca Ivers, Lisa Keay, Maoyi Tian

**Affiliations:** 1 National Centre for Non-Communicable Disease Control and Prevention, Chinese Centre for Disease Control and Prevention, Beijing, China; 2 The George Institute for Global Health, Faculty of Medicine and Health, UNSW Sydney, Sydney, Australia; 3 School of Public Health, Harbin Medical University, No. 157 Baojian Road, Nangang District, Harbin, 150081 China; 4 China Centre for Health Development Studies, Peking University, Beijing, China; 5 School of Population Health, Faculty of Medicine and Health, UNSW Sydney, Sydney, Australia; 6 School of Optometry and Vision Science, Faculty of Medicine and Health, UNSW Sydney, Sydney, Australia; 7 Department of General Practice, The Second Affiliated Hospital of Harbin Medical University, Harbin, China

**Keywords:** Falls prevention, Implementation, Participatory design, Primary health care, China

## Abstract

**Background:**

Chinese National Essential Public Health Service Package (NEPHSP) has mandated primary health care providers to provide falls prevention for community-dwelling older people. But no implementation framework is available to guide better integration of falls prevention for older people within the primary health care system.

**Methods:**

This is a two-stage online participatory design study consisting of eight workshops with stakeholders from three purposively selected cities. First, two workshops were organised at each study site to jointly develop the framework prototype. Second, to refine, optimise and finalise the prototype via two workshops with all study participants. Data analysis and synthesis occurred concurrently with data collection, supported by Tencent Cloud Meeting software.

**Results:**

All participants confirmed that the integration of falls prevention for older people within the NEPHSP was weak and reached a consensus on five opportunities to better integrate falls prevention, including workforce training, community health promotion, health check-ups, health education and scheduled follow-up, during the delivery of NEPHSP. Three regional-tailored prototypes were then jointly developed and further synthesised into a generic implementation framework by researchers and end-users. Guided by this framework, 11 implementation strategies were co-developed under five themes.

**Conclusions:**

The current integration of falls prevention in the NEPHSP is weak. Five opportunities for integrating falls prevention in the NEPHSP and a five-themed implementation framework with strategies are co-identified and developed, using a participatory design approach. These findings may also provide other regions or countries, facing similar challenges, with insights for promoting falls prevention for older people.

**Supplementary Information:**

The online version contains supplementary material available at 10.1186/s12877-024-04754-3.

## Keypoints

The integration of falls prevention for older people was weak in the Chinese PHC system.

Five opportunities were identified for better integrating falls prevention for older people in the Chinese PHC system.

We developed an implementation framework to strengthen the solid integration of falls prevention in the Chinese PHC system.

## Background

Globally, falls are ranked as the tenth leading cause of disease burden among people aged 70 years and over [1, 2]. In China, falls are the first leading cause of unintentional injury death among people aged 60 years and over and will remain as a growing public health concern with a rapid ageing population in the following decades [3]. To alleviate the anticipated burden of falls, various falls prevention programs have been implemented for older people with heterogeneous demographic and geographic characteristics since 2000 [4]. These programs demonstrated positive changes in diverse fall-related outcomes but no evidence to address long-term sustainability or scalability [4].

The challenge of sustaining and extending the benefits of effective fall-prevention programs on a large scale, particularly in resource-limited settings, was also highlighted in the recent World Health Organization guideline. It emphasized that issues such as lack of funding, infrastructure, and workforce capacity can pose significant barriers to the scalability and sustainability of fall-prevention programs. To overcome these barriers, this guideline provided several strategies, such as implementing train-the-trainer models to enhance workforce capacity, leveraging community-based organizations to expand program reach, and partnering with government and private organizations to secure funding and infrastructure [5].

Since 2009, the National Essential Public Health Service Package (NEPHSP) has been implemented in China with governmental financial support to provide essential health services to various population subgroups at the primary health care (PHC) level [6]. The number of service items in the NEPHSP expanded from nine in 2009 to 16 in 2019 [7]. A dedicated service item, named “health management service for older people”, requires PHC providers to conduct annual health check-ups and provide general health education including the prevention of falls, for community-dwelling residents aged 65 years and above [6, 8]. NEPHSP provided a great opportunity to implement falls prevention as a mandatory requirement for older people nationwide in China. However, our previous study implied that there was poor integration of fall-prevention programs for older people within the NEPHSP and identified major barriers to its implementation at the PHC level [9]. This study, therefore, aims to develop an implementation framework to strengthen the integration of falls prevention for older people within the Chinese PHC system, using a participatory design approach with a wide range of stakeholders.

## Methods

### Study setting

This qualitative participatory design study was conducted in three study sites: Chang’an District of Shijiazhuang City from Hebei Province, Beilun District of Ningbo City from Zhejiang Province, and Longhua District of Shenzhen City from Guangdong Province. The three study sites were purposively selected to represent various geographic locations and socioeconomic status. Detailed information about the three study sites has been previously reported [9].

### Study participants and sampling

Study participants were recruited from three stakeholder groups, including representatives of health administrators, public health professionals and PHC providers. Health administrators included those working at the local health commission or health bureau or Centres for Disease Control and Prevention (CDC) and in charge of the management of NEPHSP or healthy ageing-related work in the local jurisdiction area. CDC staff members who provided technical support for implementing NEPHSP were selected as public health professionals, while those who worked at the local community health centres or stations and provided falls prevention for local older people were identified as the PHC providers. All study participants were required to have worked more than one year in their organisations and were willing to sign informed consent. Participants in this study were all from the previous research. In the previous research, these participants were purposively identified from a list of potential participants developed for each study site, based on the researchers’ existing contact networks and the publicly available information from the institutional websites, prior to the recruitment. Snowball sampling was also used during the interview in the previous research to identify additional participants [9]. Emails were sent to all potential participants to confirm their willingness to participate prior to the workshop. Finally, a total of 33 participants (mean [SD] age, 41.1 [5.70] years; 14 female participants [42.4%]), including four health administrators, 13 public health professionals and 16 primary health care providers, were invited to this study. The demographic characteristics of participants are outlined in Table 1.

**Table 1:** Demographic characteristics of participants

Demographic Characteristic	Total					Chang’an district					Beilun district					Luohu district			
	Health Administrators (*N*=4)	Public Health Professionals (*N*=13)	Primary Health Care Providers (*N*=16)	Total (*N*=33)		Health Administrators (*N*=1)	Public Health Professionals (*N*=4)	Primary Health Care Providers (*N*=6)	Subtotal (*N*=11)		Health Administrators (*N*=1)	Public Health Professionals (*N*=5)	Primary Health Care Providers (*N*=5)	Subtotal (*N*=11)		Health Administrators (*N*=2)	Public Health Professionals (*N*=4)	Primary Health Care Providers (*N*=5)	Subtotal (*N*=11)
Gender (%)																			
Male	100.0%	53.8%	50.0%	57.6%		100.0%	50.0%	33.3%	45.5%		100.0%	60.0%	60.0%	63.6%		100.0%	50.0%	60.0%	63.6%
Female	0.0%	46.2%	50.0%	42.4%		0.0%	50.0%	66.7%	54.5%		0.0%	40.0%	40.0%	36.4%		0.0%	50.0%	40.0%	36.4%
Age, mean (SD), y	51.5 (2.6)	38.2 (3.3)	40.8 (4.8)	41.1 (5.7)		49 (N/A)	41.5 (1.3)	42.5 (3.1)	42.7 (3.1)		52 (N/A)	36.8 (1.9)	35.4 (1.8)	37.5 (5.1)		52.5 (3.5)	36.8 (4.0)	44 (4.2)	42.9 (6.9)
Education (%)																			
Bachelor degree and above	100.0%	69.2%	100.0%	87.9%		100.0%	50.0%	100.0%	81.8%		100.0%	60.0%	100.0%	81.8%		100.0%	100.0%	100.0%	100.0%
College diploma	0.0%	30.8%	0.0%	12.1%		0.0%	50.0%	0.0%	18.2%		0.0%	40.0%	0.0%	18.2%		0.0%	0.0%	0.0%	0.0%
Years of working (%)																			
5 years or below	0.0%	0.0%	0.0%	0.0%		0.0%	0.0%	0.0%	0.0%		0.0%	0.0%	0.0%	0.0%		0.0%	0.0%	0.0%	0.0%
5 to 10 years	0.0%	7.7%	31.3%	18.2%		0.0%	0.0%	0.0%	0.0%		0.0%	0.0%	100.0%	45.5%		0.0%	25.0%	0.0%	9.1%
10 years and above	100.0%	92.3%	68.7%	81.8%		100.0%	100.0%	100.0%	100.0%		100.0%	100.0%	0.0%	54.5%		100.0%	75.0%	100.0%	90.9%
Years of ageing-related working (%)																			
5 years or below	0.0%	30.8%	37.4%	30.3%		0.0%	0.0%	33.3%	18.2%		0.0%	40.0%	80.0%	54.6%		0.0%	50.0%	0.0%	18.2%
5 to 10 years	25.0%	61.5%	43.8%	48.5%		100.0%	75.0%	33.3%	54.5%		0.0%	60.0%	20.0%	36.3%		0.0%	50.0%	80.0%	54.6%
10 years and above	75.0%	7.7%	18.8%	21.2%		0.0%	25.0%	33.4%	27.3%		100.0%	0.0%	0.0%	9.1%		100.0%	0.0%	20.0%	27.2%

### Participatory design

Following Spinuzzi’s three-step methodology for participatory design research [10], this study consisted of two stages, including eight online workshops from December 2021 to March 2022 due to COVID-19 travel restrictions. The detailed information of the two-stage participatory design workshops were presented in Table 2.

**Table 2:** The aim, participant, step and output of the two-stage participatory design workshops in the study

Stage	Workshop	Aim	Participant	Step	Output
Stage one	The first workshop (*N* = 3)	To understand the service procedure and identify the opportunities for service integration	One workshop for one study site The number of participants in each workshop of each study site was 11	1. Introduction 2. Drawing the procedure of the health management service 3. Identifying opportunities for integrating falls prevention in the service 4. Critically reviewing and discussing all results 5. Reaching a consensus	• The procedure of health management service provided to older people • The opportunities for the integration of falls prevention in the service • Suggestions on the opportunities for integration
	The second workshop (*N* = 3)	To optimise the integration of falls prevention within the local PHC system		1. Reflecting on the suggestions about integration 2. Grouping suggestions to codes and then to themes 3. Developing a prototype of the implementation framework 4. Reaching a consensus	• Themes for integrating falls prevention • A prototype of the implementation framework • Suggestions on the opportunities for integration within the region-tailored prototype
Stage two	The first workshop (*N* = 1)	To refine participants’ understanding of how to strengthen the integration beyond the local PHC system	One workshop for three study sites The number of participants in each workshop was 33	1. Presenting and discussing findings from stage one 2. Introducing a conceptual framework from the previous study 3. Prioritising, merging and redesigning the themes across three study sites 4. Reaching a consensus	• Agreed opportunities for integration in the service • A generic implementation framework • Suggestions on the opportunities for integration within the generic framework
	The second workshop (*N* = 1)	To develop strategies for each theme in the generic implementation framework		1.Reviewing the generic framework 2. Developing and refining strategies for each theme 3. Reaching a consensus	• The implementation strategies for each theme in the generic framework

In stage one, to ensure local contexts were well considered, two workshops were carried out with participants from each study site. The first workshop aimed to understand what the health management service provided to older people and further identify the opportunities for integration of fall-prevention strategies during this service delivery as part of the NEPHSP. Integration has two meanings. First, to integrate with the NEPHSP by introducing falls prevention throughout the service cycle; Second, to integrate evidence-based interventions into the current service package, by expanding the existing content of falls prevention, limited to health education, to other intervention programs. The first workshop generated answers to three questions: First, in which phase of the health management service has falls prevention been provided to older people? Second, what form of falls prevention could be integrated into other phases of the service? Third, how can this integration be achieved? All participants were motivated to communicate and share opinions freely. At the end of the workshop, researchers and participants jointly reviewed and discussed the answers to reach a consensus. In the second workshop, a prototype of the implementation framework was jointly developed by researchers and participants, with the aim to optimise the integration of falls prevention within the local PHC system. At the beginning of the workshop, participants were invited to reflect on the suggestions on the integration from the first workshop. All suggestions were then iteratively summarised and inductively grouped to codes and then to themes by researchers in conjunction with participants. Finally, researchers and participants jointly designed the prototype of the implementation framework based on the themes.

Unlike stage one, stage two of the participatory design grouped all participants from three study sites. The first workshop aimed to refine participants’ understanding of how to strengthen the integration beyond the local PHC system. One representative from each study site was invited to present the local outputs derived from stage one, including the service procedure, opportunities for integration and the regional-tailored prototype. All participants were encouraged to provide comments after each presentation. A conceptual framework developed from the previous study with a focus on the barriers to implementing fall-prevention programs in Chinese PHC settings was introduced to all participants [9]. It was adopted to guide participants in designing a generic implementation framework by reviewing and refining the themes of three regional prototypes. In the second workshop, all suggestions on the integration emerging from previous workshops were discussed, refined and summarized to the implementation strategies for each theme by all participants.

The workshops in stages one and two were planned to last 1.5 and 2.5 h, respectively, providing participants with adequate time to express ideas and opinions without cognitive overload. The workshops conducted in this study were in line with Spinuzzi’s three steps for participatory design research. The first workshop in stage one was the initial exploration and discovery process of the participant’s work. It enabled researchers to virtually meet the participants and understand how they worked and collaborated. This, in turn, allowed both researchers and participants to reflect and think about the goals and outcomes for change. The second workshop in stage one was to develop a prototype model for change. It assisted both researchers and participants in shaping suggestions to optimize the work procedure, thus allowing for iterative improvements. Workshops in stage two, gained, adopted exactly the same methodology, to generate a generic implementation framework and strategies with a consensus. Each workshop started with an introduction or previous findings review, then gradually transited towards an open discussion process, and finally progressed to design-focused activities, including brainstorming, self-reflection and procedure mapping, aiming to form a “research and end-user partnership” in an engaging and meaningful manner. Screen sharing and digital whiteboard functions in Tencent Cloud Meeting software (Version 3.1) were used to keep participants focused, enhance the collaboration on documents in real-time and turn the online workshop into a genuine team effort.

### Data collection

All workshops were carried out by an experienced moderator (PY). The automatic transcription function in the meeting software was adopted to help the note-taker (YJ) to transcribe the recording of the discussion into texts in real time. According to Green and Thorogood’s method [11], we used thematic saturation to determine the number of workshops, which was achieved by examining whether the themes of one study site would completely differ from the themes from the other two remaining study sites and performed in the first workshop of stage two. The number of participants was determined according to Morgan’s suggestions for forming focus groups of different sizes [12]. The discussion guides were pilot tested outside three study sites to assess the consistency of researchers’ and participants’ understanding of each question. The discussion guides were iteratively refined based on the feedback from the pilot test. All discussions were conducted in Mandarin Chinese. Key information was confirmed with the participants to avoid possible misunderstandings.

### Data analysis

During the online workshop, two researchers, PY and YJ, participated in the data analysis, following the recommendations for data interpretation of Spinuzzi’s three-step methodology and Braun & Clarke’s guide [10, 13]. First, based on the advanced function of Tencent Cloud Meeting software, participants’ discussions were able to be transcribed in real-time and an online transcribed document was simultaneously generated. This allowed all participants to immediately confirm the transcribed texts via the screen or digital whiteboard. Second, with the aid of a real-time transcription function, participants summarised and articulated the key points they wanted to express in time, which facilitated the process of inductively developing the descriptive codes from the text of their discussions. Similar codes were further collated to create a theme. The generation of codes and themes was an iterative process until a consensus was met to ensure that participants’ responses could be accurately interpreted with minimum discrepancies. A conceptual framework developed from the previous study was used to facilitate the generation of the implementation framework across three study sites [9]. After the online workshop, the transcribed documents with preliminary results were sent to other two researchers (MT and YY) to seek for their suggestions. Modified results were then sent back to all participants for their final confirmation. Data coding and analysis were conducted in Chinese. The example of coding tree was provided in supplemental file 2. The consolidated criteria for reporting qualitative research (COREQ) was used to guide the reporting of this study (Supplemental File 1) [14].

### Study rigour

The rigour of this study was ensured in three aspects [15]. First, three methods were used to increase the credibility: (1) researchers (PY and YJ) participating in the data collection and analysis process had been actively engaged in falls prevention and control for older people in China; (2) different stakeholder groups were invited in this study to discuss the same topic, which could provide comprehensive information about the integration of falls prevention for older people in PHC settings; (3) key information was fed back to participants for confirmation in the workshop to avoid possible misunderstandings. Second, all quotations presented in this study were translated into English through forward-translation (from Chinese to English) and back-translation (from English to Chinese) processes to ensure the accuracy and equivalence of translated version. To maintain the confidentiality of participants, each cited quotation was marked by the participant’s role and their study identification number without any identifiable information. Third, participatory design methodology and thematic analysis guide were followed in the data coding and analysis process to minimise potential deviations or bias and guarantee conformability.

Some authors (PY, YJ, YY and LD) have rich experience in conducting community-based fall-prevention research in China, while other senior authors (RI, LK and MT) have extensive experience in designing and implementing large-scale population-based intervention programs. As all authors’ experience in conducting fall-prevention research among older people is in different contexts and settings, we constantly reflected upon the workshop to ensure that the analysis could accurately and truly reflect the concept being investigated.

### Ethics

This study was approved by the Human Research Ethics Committee, University of New South Wales (HC200418) and the Ethical Review Committee of the National Centre for Non-Communicable Disease Control and Prevention, Chinese Centre for Disease Control and Prevention (202016). Written informed consent was obtained from all participants before commencing the workshop. Consent included permission to be audio-recorded.

## Results

The average number of participants in stage one workshops was 11. The duration of these workshops was approximately 80 to 130 min (median [inter-quartile range], 92.5 [90–100] minutes). All participants taking part in stage one workshops also participated in stage two workshops, which lasted about 160 min each.

### Opportunities for integration

**Fig. 1:**
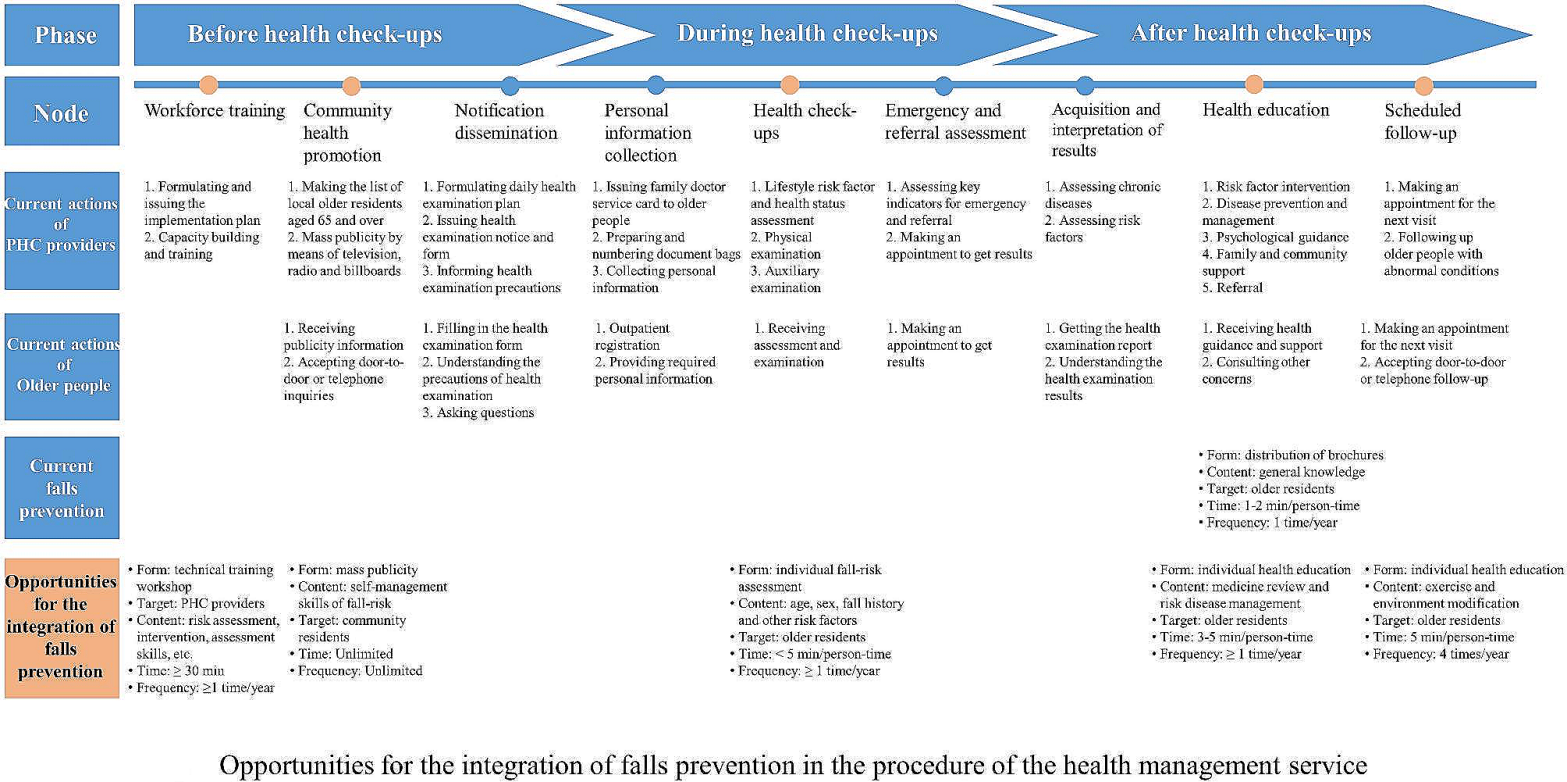
Opportunities for the integration of falls prevention in the procedure of the health management service

The procedure of the health management service for older people in the NEPHSP slightly varied across three study sites. As all age-eligible people were provided with annual health check-ups, participants concurred on dividing this service procedure into three phases, before, during, and after the health check-ups, encompassing nine key nodes. The first three key nodes of phase one were the publicity and mobilization work in preparation for health check-ups. The second three key nodes of phase two were the personal information collection, health check-ups and emergency and referral assessment. The last three key nodes of phase three were the acquisition and interpretation of the results, health education and scheduled follow-up. Detailed information on the service procedure is presented in Fig. 1.

All participants recognised that falls prevention was currently only available in the health education node of phase three of the health management service, which is completely disconnected from the previous two phases. Participants agreed that there could be five opportunities for integrating information and actions on why and how to prevent falls based on individual risk of falls at three phases in the service **(**Fig. 1**)**. In phase one, PHC providers would be beneficial from receiving dedicated technical training on implementing falls prevention, including risk screening, fall-prevention needs assessment and tailored intervention provided to the older people in the workforce training node. The community health promotion node serves as a good opportunity to convene health campaign programs to increase the awareness of falls prevention among older residents. In phase two, the data closely associated with the risk of falls collected from the health check-ups node, e.g., history of diseases, medications utilisation, body mass index, vision, hearing, activities of daily living, and motor function, could be used to assess the individual risk of falls. This information could also be used to guide the development of individually tailored interventions for older residents, e.g., specific medication review and withdrawal or multimodal exercise interventions, in the health education and scheduled follow-up node of phase three. Suggestions on home modifications to remove environmental hazards could be provided for older people in the last node of phase three.

“*The current health education was too general. It could be tailored according to the physical examination results of the older people, especially for those with hypertension and diabetes.*” (CDC staff 10201).

### Region-tailored prototype development

**Fig. 2:**
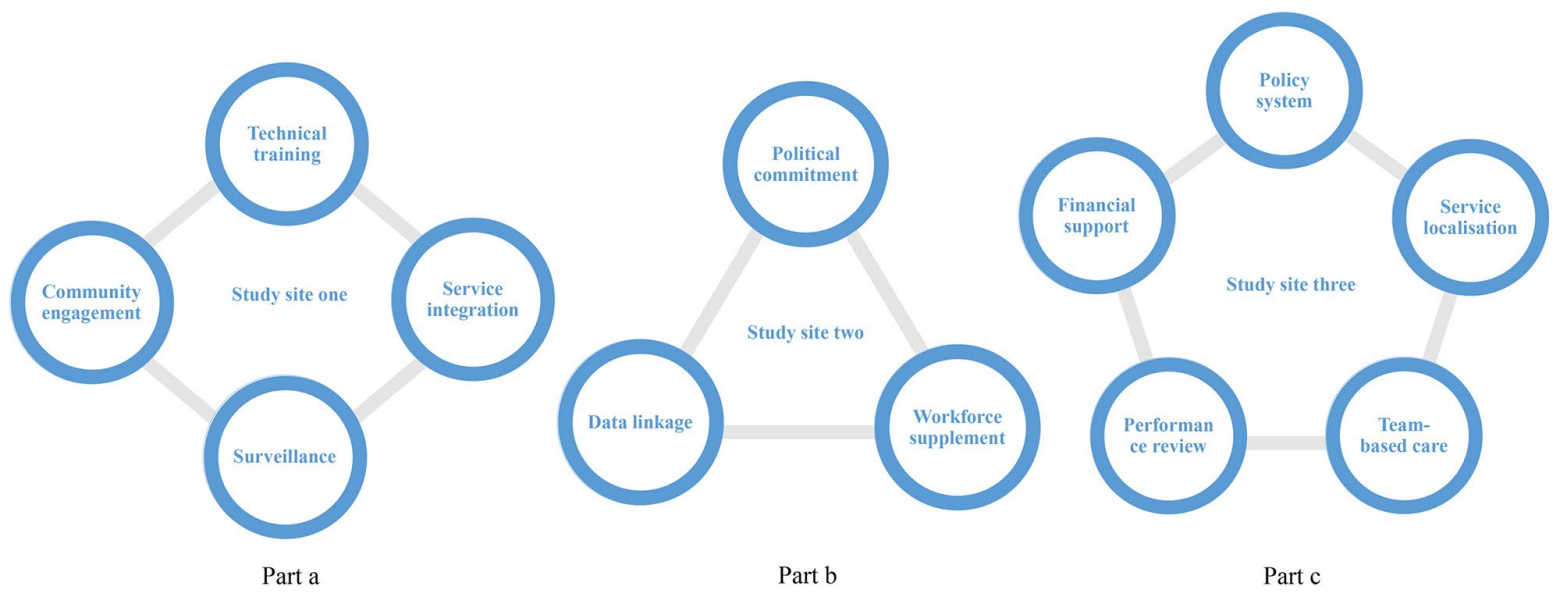
Visualisation of three region-tailored prototypes

Followed by service procedure mapping, participants from each study site further developed a regional-tailored implementation framework **(**Fig. 2**)**. In study site one, participants constructed a four-component prototype including surveillance, technical training, service integration and community engagement **(Part a**, Fig. 2**)**. Specifically, surveillance, to use data-driven solutions to guide the development of fall-prevention interventions; Technical training, to provide on-the-job training for PHC providers to acquire professional knowledge and improve skills in implementing fall-prevention interventions; Service integration, considering diabetes and hypertension as risk factors for falls, participants suggested that fall-prevention activities can be integrated with the management of diabetes and hypertension. Both are also service items within the NEPHSP. Lastly, participants urged the transition of fall-prevention intervention from a disease-based model towards a people-centred model through community engagement.

*“The information of injurious falls, such as the severity, treatment and outcomes, were captured in the hospitals. We should use this information to inform the decision-making of falls prevention for residents.” (CDC staff 10203).*

“*When we conducted fall-prevention health education, older people always raised questions about their other health conditions. The management of fall-related diseases might promote their interest and compliance in falls intervention.*” (PHC provider 10302).

Participants from study site two formed a three-component prototype comprising data linkage, workforce supplement and political commitment **(Part b**, Fig. 2**)**. They emphasised the linkage with health insurance data to support the fall-prevention decision-making process. They widely perceived that the shortage of PHC providers in the follow-up of older residents can be potentially task-shifted by trained non-professional healthcare workers. They were in complete agreement that political commitment is critical in falls prevention, one of the examples was to include falls prevention as part of the regional action plan on healthy ageing.

“*If falls prevention for older people could be included in the regional work agenda, we could obtain stronger support from local government departments, particularly in human resources and finance.*” (Health administrator 20101).

“*Some civil organizations dedicated themselves to enhancing the well-being of older people, and their staff and volunteers were willing to participate in falls intervention.*” (PHC provider 20305).

A prototype was built upon five components by participants from study site three, i.e., performance review, team-based care, financial support, service localisation and policy system **(Part c**, Fig. 2**)**. They argued that combining quantitative results of interventions and qualitative feedback from older people could better assess PHC providers’ work performance. They strongly emphasised that a team-based care model involving multi-disciplinary professionals, such as geriatricians, pharmacists, nurses and dieticians, can strengthen the current PHC workforce. To scale up evidence-based home modification and exercise interventions for older residents, participants suggested making use of previously untapped resources from the local private sectors to increase the financing base through a public-private partnership. Given the long history of traditional Chinese medical practices in study site three, Taichi and Baduanjin were suggested as prescriptions in the Traditional Chinese Medicine service item within the NEPHSP. Similar to study site two, participants also conceived the idea of establishing a consolidated policy system on healthy ageing to gain policy support for falls prevention, from diverse government departments.

“*Some private sectors with interest in promoting the health of older people could be actively engaged to provide supplementary financial support for some expensive programs, for example, the assessment and modification of environmental hazards at home.*” (PHC provider 30305).

“*Using dialects for health education would be more welcoming for older people. Including local cultural elements in current services would likely appeal to more older people.*” (Health administrator 30102).

### Generic framework synthesis

**Fig. 3:**
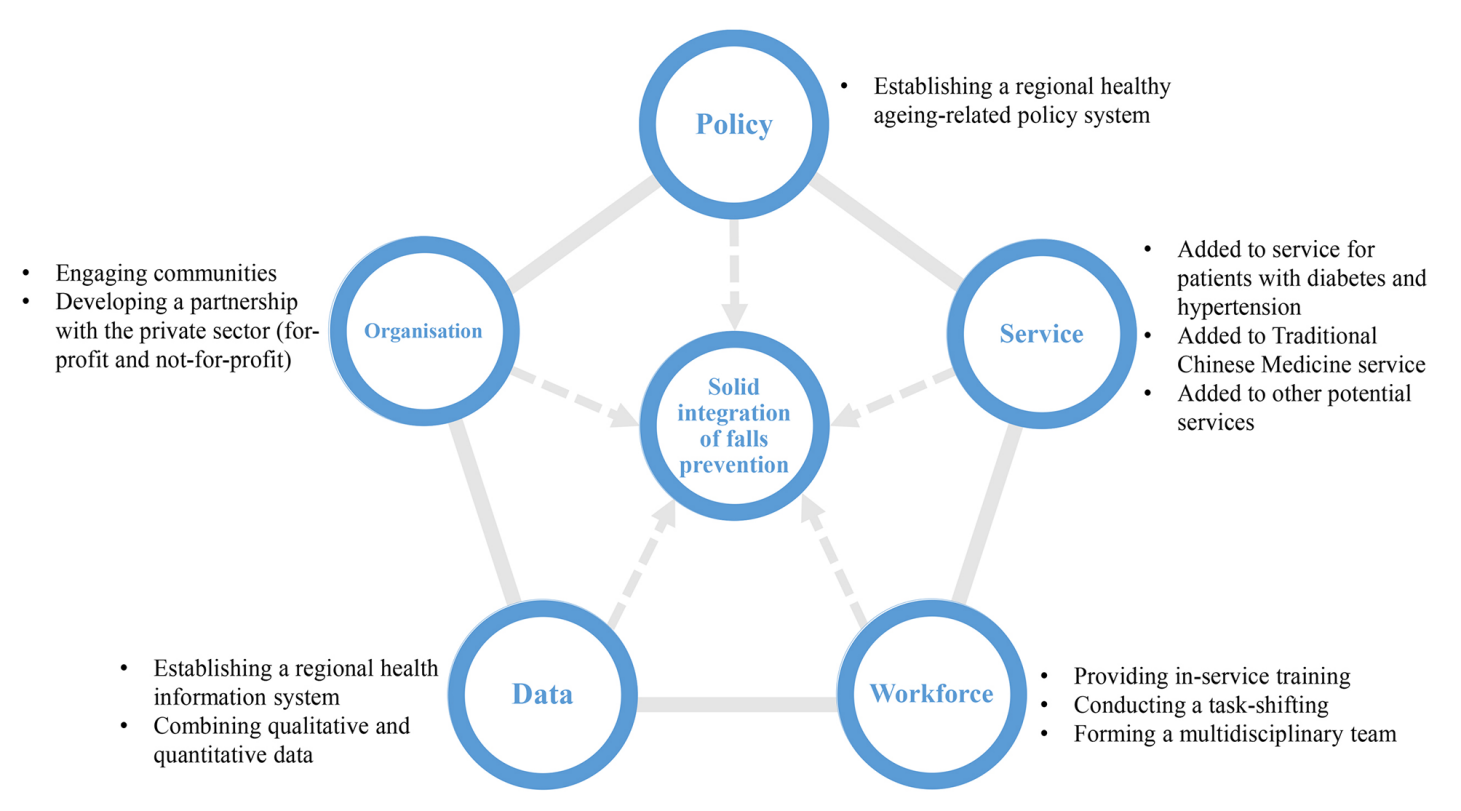
Visualisation of the implementation framework

A generic framework was derived from similar or interrelated themes across three regional-tailored prototypes **(**Fig. 3**)**. First, all three study sites highlighted the essential role of data in understanding the burden of falls, supporting decision-making, developing interventions and tracking work performance. Data was, therefore, identified as a common theme combing surveillance, data linkage and performance review. Second, the workforce was generated as another common theme in all local prototypes, consisting of PHC provider training, involving non-professional health workers and forming team-based coordinated care. Third, service integration and service localisation in study sites one and three were merged into the service theme. Fourth, community engagement and financial support from private sectors produced from study sites one and three were synthesised into the organisation theme. Both address the promise of external organisations participating in falls prevention. Lastly, political commitment and policy system emerged from study sites two and three underscored the importance of policy support, and thus combined into the policy theme. The five themes interact with each other and do not necessarily occur sequentially.

### Implementation strategies development

There were 11 strategies developed across the five themes **(**Fig. 3**)**. Specifically, the data theme advocates using a broad range of data sources to support decisions in reprioritizing resources for falls prevention among older people. It contained two strategies. First, the local government or health administrative department should establish a regional integrated health information system to regularly synthesize multi-source heterogeneous data from diverse institutions to quantify the health and economic burden of falls among local older residents and support the design, implementation and adaption of fall-prevention interventions. Second, in combination with these quantitative health outcomes, PHC providers should regularly collect qualitative feedback data from older residents to yield more valuable insights to drive performance improvement. The workforce theme aims to improve the availability and quality of health workers engaged in falls prevention for older people. Participants suggested three strategies to achieve this theme. First, the local health administrative department and PHC institutions should institutionalize in-service training and continuing education for current PHC providers, particularly for new staff, to upgrade their professional knowledge and skills in falls prevention. Second, the local health administrative department, in collaboration with the human resources department, should identify additional workforce resources, e.g., community health advisors, social service workers, peer health educators and volunteers, to assist current PHC providers in better responding to evolving health needs of older residents and extend current intervention reach, particularly for individuals not normally engaged in interventions due to language or cultural factors. Third, health governors should consider a strategic redistribution of work among current PHC providers to form a multidisciplinary team in which all members could share responsibilities and play an integral role in providing older residents with fall-prevention interventions.

The organisation theme expects to gain broader public support for the design and implementation of fall-prevention interventions through the early and active engagement of diverse stakeholder groups. PHC providers should create honest conversations, reciprocal relationships and mutual trust for older residents to mobilize their capabilities of fall-risk identification and management. PHC institutions should create a sustainable partnership with the private sector, e.g., civil society organisations and commercial companies, to serve the diverse fall-prevention needs of the older population. The service theme highlights the interlinkage of different services in NEPHSPH that could potentially facilitate the effectiveness of fall-prevention interventions for older people. Local health governors could integrate falls prevention into the regional work plan of health management services for diabetes and hypertension to better serve older patients with these diseases and also add Chinese-style exercises, e.g., Taichi and Baduanjin, to the Traditional Chinese Medicine service. The policy theme strives to require stronger and concerted political commitment and response to falls prevention for older people from the top levels of government. The local health administrative department should collaborate with other government departments to establish a consolidated policy system at the regional level to sustainably and coherently support falls prevention for older people.

## Discussion

This participatory design study identified five opportunities, workforce training, community health promotion, health check-ups, health education and scheduled follow-up, to consistently provide older people with fall-prevention services in the health management service of NEPHSP. It also developed a generic five-themed implementation framework with 11 strategies by merging three regional-tailored prototypes to strengthen the integration of falls prevention for older people within the Chinese PHC system.

Despite there being established data to record fatal and non-fatal outcomes of falls from multiple sources [16, 17], the information system remains fragmented mainly due to heterogeneous data structures, inconsistent management mechanisms, unsound security regulations, and poor interoperability [18, 19]. Establishing a regional integrated health information system was identified as a key strategy to facilitate the synthesis, management and utilisation of the multi-source data. This integrated data system could be used to assess disease burden and monitor performance. For example, de-identified individual information could be aggregated at the population level to depict the burden of falls among older people in terms of incidence, prevalence and death. Data across periods of the same individuals could also be linked to monitor the long-term health loss and economic burden caused by falls. Previous studies demonstrated the value of similar platforms in tracking performance and supporting the data-driven policymaking process to combat other health challenges [20, 21]. Following the above quantitative analysis, an open-ended qualitative survey could be performed to glean insights from the emotional responses and rational thoughts of older people on fall-prevention interventions. Previous studies reported that using a mixed methods approach could help PHC decision-makers and providers better understand the impacts of diverse factors on sustaining intervention activities and identify contextually appropriate solutions to address the barriers [22].

The challenges in the supply, distribution and quality of the health workforce in Chinese PHC settings are major barriers in the implementation of fall-prevention interventions for older people [23–25]. Participants jointly identified in-service training as an evidence-based strategy in the workforce theme to address this concern. In-service training could be designed as a series of in-person workshops or e-learning courses for individuals or teams, for example, hands-on training on fall-risk assessment questionnaires and scales, in-person group training courses for communication skills with older people and team building to cultivate cohesiveness among members. Previous studies showed that in-service training could help healthcare providers to improve their professional abilities in data management and clinical treatment [26–28]. Chinese Central Government strongly advocated the establishment of medical alliances in recent years [29]. This mechanism refers to that different types of health and medical institutions in a certain region or across regions are integrated as a joint organisation. Within this organisation, PHC institutions could refer patients with severe and complicated diseases to high-level hospitals [29]. High-level hospitals are required to drive the sustainable development of PHC institutions through technical support and personnel training [29]. This mutually beneficial relationship lays a solid foundation for PHC providers to receive regular in-person training by professionals from different disciplines. In addition, self-paced online courses were shown effective in improving the professional knowledge and behaviour of PHC providers in infectious disease management [30, 31]. Chinese Continuing Medical Education Network, as the national continuing education provider [32, 33], could be a good platform to realise the large-scale self-paced online learning activities of falls prevention for older people among PHC providers in different forms, including experience sharing, case-based learning, practical skill teaching and clinical simulation.

Harnessing the potential of non-professional health workers was proposed as the second viable strategy in the workforce theme to address the gaps of formally employed and trained health workers. For example, some older residents willing to put in extra effort to support the implementation of NEPHSP could be selected as volunteers and then trained to be community health advisors in association with the local PHC system. As community members, they could have identities that are more similar to other older residents in terms of age, socioeconomic status and life experience and have more opportunities to reach an optimal peer connection with target older community groups. They could share falls experiences and impart reliable prevention messages to their peers in an acceptable and respectful fashion. They could also provide their audiences with demonstrations on how to identify and manage simple fall risk factors and persuasive suggestions for seeking specialist services for more complex risk factors. Previous studies demonstrated the positive impacts of lay health workers in alleviating the shortage and disproportionally distribution of professional health workers and improving primary and community health care [34–36]. In addition, effective fall-prevention interventions always require a combination of various skills and knowledge from a multidisciplinary team, rather than simply distributing brochures, to improve safe mobility for older adults [5]. For example, rehabilitation therapists could create custom exercises to address identified deficits of lower body strength, flexibility, and balance. Podiatrists could assess the gait and provide suggestions on selecting footwear that could provide support, fit properly and are non-slip. Optometrists could provide appropriate visual acuity assessment and correction to minimise the visual risk of falling. The multidisciplinary team approach proposed as the third strategy in the workforce theme has been widely proven to be effective in improving people-centred continuous management and control of different health priorities and reducing workloads and burnout of health service providers [22, 37, 38].

In the organisation theme, community engagement was highlighted as the first strategy to empower older people to manage their fall-risk factors from awareness-raising to action adoption. Being equipped with a positive awareness, e.g., falls are a preventable health issue rather than an inevitable consequence of ageing, older people could be motivated to actively express their concern or fear of falling, share the experience of previous falls and seek appropriate suggestions from the PHC providers and, in turn, enable PHC providers to have a deeper understanding of fall-prevention needs among older residents. Additionally, some private sectors have a common interest with PHC institutions in reducing the hazards of falls and promoting the health of older people. An open and transparent communication mechanism could be established to initialise equal dialogues, seek mutually beneficial opportunities and foster extensive involvement in the decision-making process between PHC institutions and the potential private sector. This second strategy could lay a solid foundation for the collaborative partnership to harness the expertise, information, and resources of the private sector to scale up the implementation of falls prevention for a large older population, particularly those evidence-based environmental modifications requiring substantial financial affordability [5].

There have been two services, the health management service for patients with hypertension and type 2 diabetes, closely related to the health of older people in the NEPHSP since 2009 [6, 7]. Previous studies demonstrated that diabetes complications, e.g., retinopathy and peripheral neuropathy, significantly contributed to gait instability and falls among older people [39]. There was also evidence that hypertensive older adults had a higher prevalence of falls [40]. However, falls prevention was completely ignored in these two services [6, 7]. Therefore, in the service theme, participants suggested that disease-specific fall-prevention interventions could be developed and added to the two services. This change could benefit more older people, particularly those at higher risk of falls, and also facilitate integrated care for older people in the PHC settings. A similar change could be adopted in the Traditional Chinese Medicine (TCM) service added to the NEPHSP in 2017. Previous studies reported a range of health benefits of Taichi and Baduanjin as traditional Chinese Qigong exercises, particularly in maintaining the balance, strength, and flexibility of older people [41–44]. Participants strongly supported the integration of these two Chinese-style exercises into the TCM service, which was proven to effectively reduce the risk of falls and benefit the holistic health status of older people in the PHC settings [42, 43].

There was a lack of national policy support for falls prevention [45]. Participants agreed that it could be possible for regional policies to take a step forward in falls prevention, particularly in those areas with a high burden of falls among older people. Establishing a regional healthy-ageing-related policy system was identified as the most critical and fundamental approach in the policy theme to ensure the sustainable development of falls prevention for older people. To achieve this goal, champions from local health sectors could first identify key decision-makers in other government departments related to the health of older people through stakeholder analysis and understand the dynamic relationship among those stakeholders through observation and interview. Then, a policy brief could be adopted to inform key decision-makers of the importance of falls prevention as a particular issue in the perspective of their accountabilities and provide them with possible policy options and recommendations. This process could drive some potential decision-makers to put falls prevention for older people on their political agenda or provide explicit policy support in their policy domains related to the health of older people. Political commitments from diverse policy domains could facilitate the formulation of a policy system across sectors as a whole-of-government approach to systematically address the broader determinants of falls, including socioeconomic, cultural and environmental factors, and improve overall societal gains in population health and health equity [5].

This study had four limitations. First, the purposive sampling method of study sites and participants constrained the generalizability of findings in this study. However, readers and potential users could use detailed information on the method in this study to assess the transferability of these findings in other PHC settings. Second, because of COVID-19, it was difficult to organize online workshops with older people, who had low digital literacy. This concern could be partly alleviated by interviewing participants with extensive work experience, who would share similar feedback and reflections of older people. Third, this study was designed and conduced based on the prior research. Researchers know and had existing connections with the study participants. This may influence the study outcomes. To reduce potential biases, researchers were required to constantly reflect upon their attitudes, assumptions and positions in the co-design process, to ensure that their understanding could truly reflect the participants’ perspectives. Fourth, the implementation framework with strategies should be validated and triangulated in the future with quantitative data analysis.

## Conclusion

NEPHSP has provided a great opportunity to deliver falls prevention for Chinese older people since 2009. However, this study confirmed a weak integration of falls prevention in this government-led work, identified five opportunities for integrating falls prevention into the health management service, and further developed an implementation framework with 11 strategies to achieve a solid integration of falls prevention in the Chinese PHC system. These findings could also bring other regions or countries some insights or references for promoting the health of older people in similar PHC settings within an ageing context.

### Electronic supplementary material

Below is the link to the electronic supplementary material.

Supplementary Material 1

Supplementary Material 2

## Data Availability

The de-identifiable data presented in this study are available on request from the corresponding author. The data are not publicly available due to privacy restrictions.

## Abbreviations

NEPHSP: National Essential Public Health Service Package
PHC: primary health care
COREQ: consolidated criteria for reporting qualitative research
CDC: Centres for Disease Control and Prevention
COVID-19: Corona Virus Disease 2019
