# Supplementary material for: Using a participatory design to develop an implementation framework for integrating falls prevention for older people within the Chinese primary health care system
Source: BMC Geriatr. 2024 Feb 21;24:178. doi: 10.1186/s12877-024-04754-3 (PMC10882749; doi:10.1186/s12877-024-04754-3)
Supplement: Supplementary file 1 — Supplementary Material 1 [file 12877_2024_4754_MOESM1_ESM.docx]

Supplemental file 1

Table 1 COREQ (COnsolidated criteria for REporting Qualitative research) Checklist (for the submmitted version)

*The reported line number and page number correspond to the clean version of the manuscript uploaded as a related file

| **Topic** | **Item No.** | **Guide Questions/Description** | **Reported on**  **Page No.** |
| --- | --- | --- | --- |
| **Domain 1: Research team and reﬂexivity** | | | |
| *Personal characteristics* | | | |
| Interviewer/facilitator | 1 | Which author/s conducted the interview or focus group? | Page 11, Lines 18-19 |
| Credentials | 2 | What were the researcher’s credentials? E.g. PhD, MD | Page 1, Lines 6-7 |
| Occupation | 3 | What was their occupation at the time of the study? | PY, YJ, LD (China CDC staff);  YJ (PhD candidature at UNSW);  YY (Senior researcher at PKU);  RI, LK (Professor at UNSW);  JP (Student at HMU);  MT (Professor at HMU)  Page 1, Lines 8-17 |
| Gender | 4 | Was the researcher male or female? | YJ, RI, LK, LD (female); PY, JP, YY, MT (male)  Not considered to affect the study |
| Experience and training | 5 | What experience or training did the researcher have? | YP, JY, JP (qualitative research training); MT (rich experience in qualitative research); YY (rich experience in geriatrics); YP, JY, RI, LK, LD, MT (rich experience in injury prevention) |
| *Relationship with participants* | | | |
| Relationship established | 6 | Was a relationship established prior to study commencement? | Page 27, Lines 10-15 |
| Participant knowledge of  the interviewer | 7 | What did the participants know about the researcher? e.g. personal  goals, reasons for doing the research | The goal and reason of this research have been articulated in the written informed consent.  Page 14, Line 9-11 |
| Interviewer characteristics | 8 | What characteristics were reported about the interviewer/facilitator?  e.g. Bias, assumptions, reasons and interests in the research topic | Falls prevention is not well-integrated in the National Essential Public Health Service Package.  Page 7, Lines 6-8  Page 13, Line 22-Page 14, Line 3 |
| **Domain 2: Study design** | | | |
| *Theoretical framework* | | | |
| Methodological orientation and Theory | 9 | What methodological orientation was stated to underpin the study? e.g. grounded theory, discourse analysis, ethnography, phenomenology,  content analysis | Participatory design  Page 8, Line 25  Page 10, Line 23-Page 11, Line 8 |
| *Participant selection* | | | |
| Sampling | 10 | How were participants selected? e.g. purposive, convenience, consecutive, snowball | Page 8, Lines 8-15 |
| Method of approach | 11 | How were participants approached? e.g. face-to-face, telephone, mail, email | Page 8, Lines 8-15 |
| Sample size | 12 | How many participants were in the study? | Page 8, Lines 15-18 |
| Non-participation | 13 | How many people refused to participate or dropped out? Reasons? | None of the participants refused. |
| *Setting* | | | |
| Setting of data collection | 14 | Where was the data collected? e.g. home, clinic, workplace | Online.  Page 12, Lines 8-25 |
| **Topic** | **Item No.** | **Guide Questions/Description** | **Reported on**  **Page No.** |
| Presence of non-  participants | 15 | Was anyone else present besides the participants and researchers? | None. |
| Description of sample | 16 | What are the important characteristics of the sample? e.g. demographic data, date | Table 1 |
| *Data collection* | | | |
| Interview guide | 17 | Were questions, prompts, guides provided by the authors? Was it pilot tested? | Page 9, Lines 18-21  Page 12, Lines 1-3 |
| Repeat interviews | 18 | Were repeat interviews carried out? If yes, how many? | No repeated interviews. |
| Audio/visual recording | 19 | Did the research use audio or visual recording to collect the data? | Page 14, Lines10-11 |
| Field notes | 20 | Were ﬁeld notes made during and/or after the interview or focus group? | Page 11, Lines 8-15  Page 12, Lines 8-25 |
| Duration | 21 | What was the duration of the interviews or focus group? | Page 14, Lines 14-17 |
| Data saturation | 22 | Was data saturation discussed? | Page 10, Lines 7-17  Page 11, Lines 20-24 |
| Transcripts returned | 23 | Were transcripts returned to participants for comment and/or correction? | Page 12, Lines 13-14  Page 12, Lines 24-25 |
| **Domain 3: analysis and ﬁndings** | | | |
| *Data analysis* | | | |
| Number of data coders | 24 | How many data coders coded the data? | Page 11, Lines 12-15  Page 12, Lines 8-9 |
| Description of the coding  tree | 25 | Did authors provide a description of the coding tree? | Supplemental file 1 |
| Derivation of themes | 26 | Were themes identiﬁed in advance or derived from the data? | Page 10, Lines 2-4  Page 10, Lines 15-19 |
| Software | 27 | What software, if applicable, was used to manage the data? | Page 11, Lines 12-13 |
| Participant checking | 28 | Did participants provide feedback on the ﬁndings? | Pages 9-11 |
| *Reporting* | | | |
| Quotations presented | 29 | Were participant quotations presented to illustrate the themes/ﬁndings?  Was each quotation identiﬁed? e.g. participant number | Page 16, Lines 4-6  Page 16, Line 22-Page 17, Line 3  Page 17, Lines 16-21  Page 18, Lines 13-19 |
| Data and ﬁndings consistent | 30 | Was there consistency between the data presented and the ﬁndings? | Pages 14-18 |
| Clarity of major themes | 31 | Were major themes clearly presented in the ﬁndings? | Fig.2  Fig.3 |
| Clarity of minor themes | 32 | Is there a description of diverse cases or discussion of minor themes? | No minor theme. |

Developed from: Tong A, Sainsbury P, Craig J. Consolidated criteria for reporting qualitative research (COREQ): a 32-item checklist for interviews and focus groups. International Journal for Quality in Health Care. 2007. Volume 19, Number 6: pp. 349 – 357


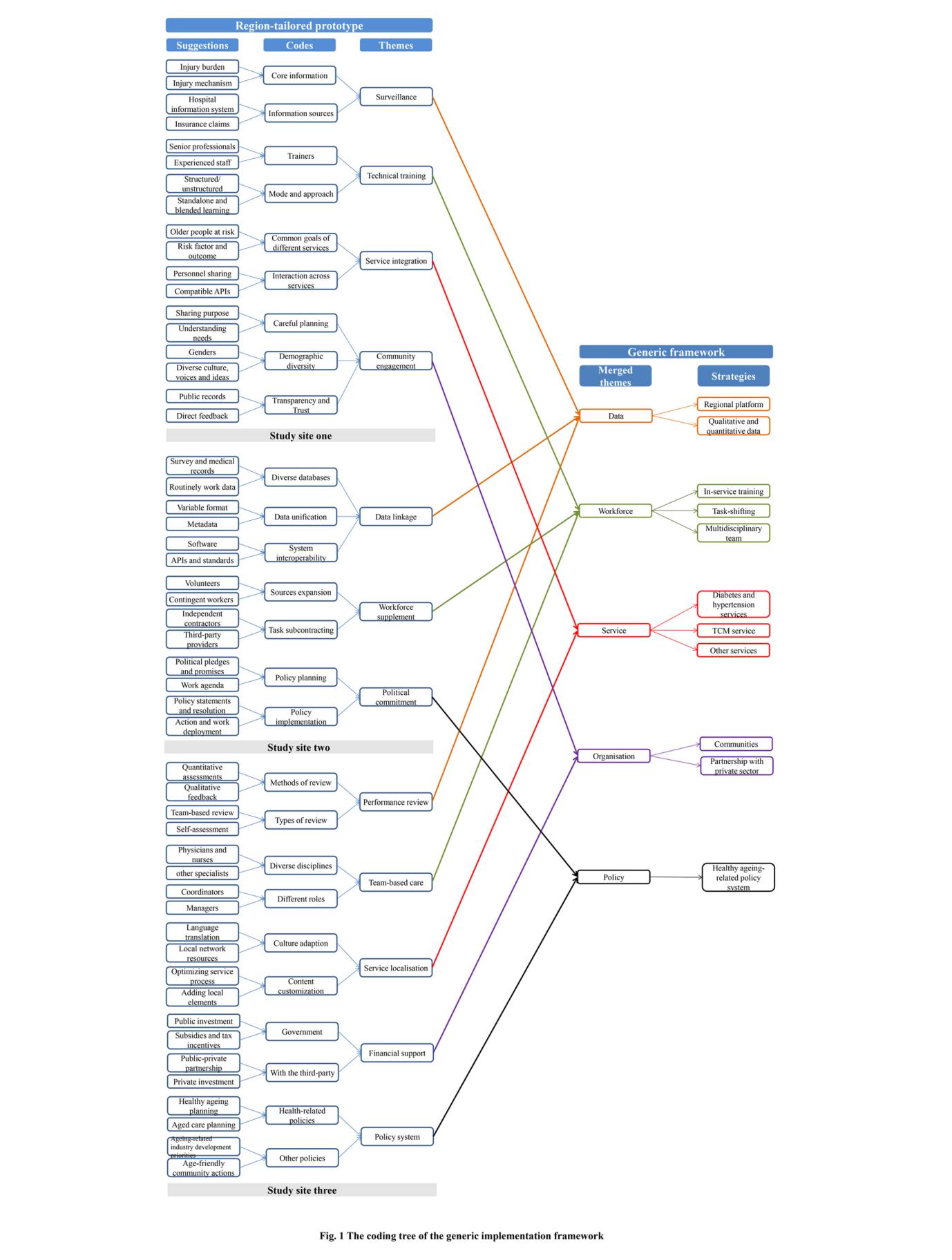


Fig. 1 The coding tree of the generic implementation framework
