# Supplementary figures and images for: Using a participatory design to develop an implementation framework for integrating falls prevention for older people within the Chinese primary health care system
Source: BMC Geriatr. 2024 Feb 21;24:178. doi: 10.1186/s12877-024-04754-3 (PMC10882749; doi:10.1186/s12877-024-04754-3)

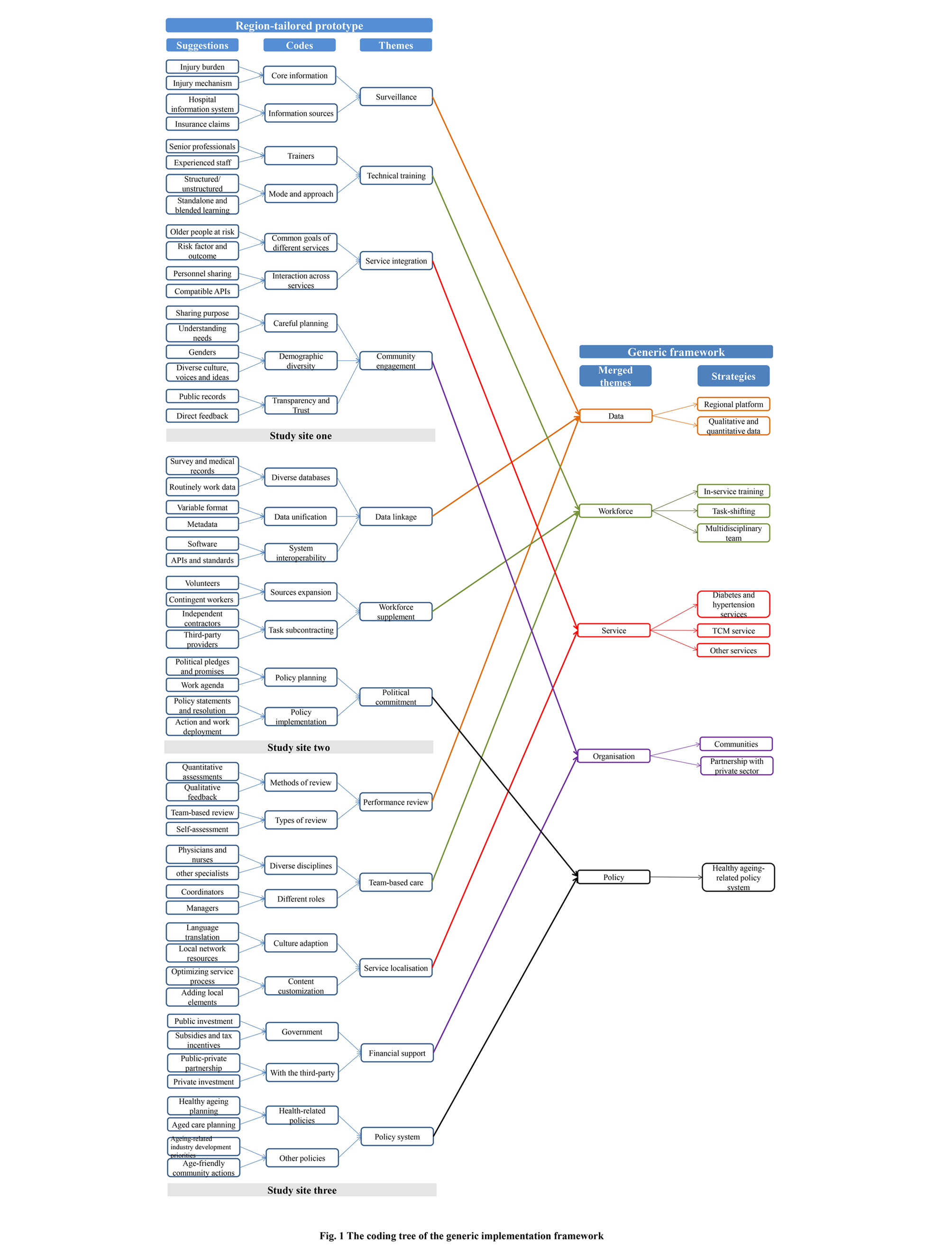

Supplement: Supplementary file 2 — Supplementary Material 2 [file 12877_2024_4754_MOESM2_ESM.jpg]
